# Impact of sugar beet seed priming using the SMP method on the properties of the pericarp

**DOI:** 10.1186/s12870-020-2246-4

**Published:** 2020-01-20

**Authors:** C. Chomontowski, S. Podlaski

**Affiliations:** Department of Plant Physiology, Institute of Biology, Warsaw University of Life Sciences SGGW, 159 Nowoursynowska St, 02-776 Warsaw, Poland

**Keywords:** Porosity, Density, Water content, Water potential, Germination

## Abstract

**Background:**

This study determined the effects of two solid matrix priming methods on changes in the characteristics of two lots of the same variety of sugar beet fruits that differ in the level of vigour.

**Results:**

Seed treatment within each level of vigour did not significantly affect helium and apparent density, total pore volume and total porosity. However, there was a tendency to increase porosity due to priming. This is probably why seed priming significantly increased mesopore diameter in both high and low vigour seeds. These changes increased the water content in the pericarp and the seeds and increased the water potential during germination. The high level of electrical conductivity of the fruit extracts was associated with low seed vigour. Low vigour resulted in higher humidity of the pericarp and decreased seed moisture and was also associated with lower water potential of the pericarp and seeds.

**Conclusions:**

A significant difference in the water content in the pericarp and seeds was indicative of imbibition and problems with water flow between these centres, which resulted in a low water diffusion coefficient of the pericarp. This low water diffusion coefficient was correlated with the prolongation of the seed germination time.

## Background

Water availability is one of the basic factors determining the seed germination process. Moreover, water availability is determined by the properties of the germination medium and by the morphological, anatomical and physicochemical properties of the fruits. The most important environmental factors include the gradient of water potential, the water transport parameters in the germination medium and the conductivity and diffusivity of the soil [12].

A sugar beet fruit is composed of a hard pericarp, whose mass varies from 70 to 80% [22, 33] of the raw fruit. Inside the pericarp, there is a kidney-shaped botanical seed [2, 8, 13, 14, 19]. The beet pericarp can restrict the water and oxygen uptake of an enclosed seed [8, 13, 18, 25, 27, 30, 31, 34]. The upper part of the pericarp is covered with an ovary cap (operculum), on which the remnants of the stigma are placed. Sometimes there is a hole that remains in the ovary cap after the transmission channel [27]. On the opposite side of the operculum, there is a place where the fruit attaches to the shoot, which is called the basal pore. The basal pore is probably the most important place for water to flow to the seed [2, 8, 14, 25]. The structure of the pericarp, the size of the basal pore and its degree of tissue filling are genetically determined by the maternal genotype.

SEM analysis of fruits from 48 Polish sugar beet lines *has shown, that a* beet pericarp consists of three layers [28]. The first layer in the vicinity of the seed cavity is made of small sclereids with thick cell multi-layer walls. Large, single crystals of chemical compounds are present in this layer. The middle layer of the pericarp is made of sclereids with thinner cell walls. Inside these sclereids, there are clusters of numerous small crystals of chemical compounds. The second layer of the pericarp gradually passes into the third layer, which is made of parenchyma cells. However, in the fruit of some commercial varieties it is difficult to separate two layers of sclerenchyma tissue.

The pericarp thickness in the basal pore ranges from 0.6 to 0.96 mm [27]. The ratio of the pericarp parenchyma layer thickness to the sclerenchyma layer thickness determines the density, water potential and water flow through the pericarp. The pericarp density varies from 0.56 to 1.10 g cm^− 3^ [27]. Because parenchyma is loose tissue and sclerenchyma is compact and dense, the thicker the sclerenchyma tissue is in relation to the thickness of whole pericarp (e.g., as a result of fruit polishing), the higher the density of the pericarp and the lower the general porosity and water potential of the pericarp are at a given time.

X-ray analysis of the chemical compound crystals showed that they include the following elements: potassium, calcium, magnesium, phosphorus, chlorine and sulphur. Based on the analysis of fruit water extracts, potassium, sodium [15] magnesium and calcium are predominant among the cations, whereas nitrate, chloride, phosphate and sulphate oxalate [16] are predominant among the anions [18]. Crystals dissolve in water during seed imbibition, which results in the formation of a solution with a low osmotic potential and a high electric conductivity in the pericarp [26]. This solution inhibits the water flow through the pericarp, which is reflected in the low pericarp water diffusion coefficient [27].

Hadas [12] and Blunk et al. [3] point out that water flow through pericarp or seed coat is important for seed germination. One of the measures of water flow is the water diffusion coefficient. Podlaski [27] assessed the value of the pericarps water diffusion coefficient in raw fruits originating from 48 sugar beet breeding lines reproduced in Poland. The average water diffusion coefficient of the pericarp during the germination period was 0.00134 cm^2^ d^− 1^ [27]. Seed coat water diffusion of chickpea, pea, and vetch ranged from 0,03 to 0,00009 cm^2^ d^− 1^. The lower values were for low seed coat hydration [12].

In addition to the inorganic compounds of osmotic character in the pericarp, many organic compounds have been identified: vanillic acid, p-oxybenzoic acid, ferulic acid, coumarin acid, chlorogenic acid, ABA, rutin and protocatechuic acid [10, 13, 14, 30, 31] Interestingly, levels of several endogenous plant growth regulators, which were shown to influence the germination or early root growth, greatly differed between the pericarp and the true seed. Consequently, the pericarp is assumed to play an important role during the germination and seedling growth of sugar beet [1]. There is a lack of information regarding whether these germination-inhibiting compounds affect the flow of water through the pericarp.

There is also no obvious answer to the question of whether the water penetrates the pericarp through the whole surface or whether there are special flow points (pores), i.e., points of entry. Chachalis and Smith [6] showed that the presence of a high density of deep and open pores in a soybean seed coat was connected with the rapid permeability of the seed coat. According to Manz et al. [20], the micropylar tobacco seed end is the major entry point of water. The research of Juntilla [18] and Podlaski [27], who covered the base of the fruit, the top of the operculum and the surfaces around it with a silicone paste, showed that the main point of water entry might be the basal pore.

The low water potential of the pericarp causes a reduction in the flow of water to the seed, creating a kind of barrier. The water potential of seeds just before the emergence of the germinal root is approximately − 1.0 MPa [4, 27], therefore any environmental factor or seed property that delays the achievement of such a water potential level prolongs the germination process [29].

To improve the germination of sugar beet seeds, priming processes are commonly used. The physiological effects of priming (increased germination and emergence speed) are well known [7, 11, 21, 24]. However, there is a lack of knowledge about whether and to what extent the effects of priming depend on changes in the properties of pericarp to increase the flow of water. This study attempts to answer this question.

## Methods

Throughout the paper, the term “fruit” refers to the sugar beet dispersal unit, and “seed” refers to the botanically true seed and includes the embryo, perisperm, and endosperm and seed coat remnants.

Seeds of *Sugar beet* were collected from plants in Cesena town, Forlì-Cesena county, Romagna province, Italy (Longitude (E) 12° 15′ 44.6″; Latitude (N) 44° 09′ 59.8″; Altitude 25 m). As *Sugar beet* spp. are not endangered, collection of samples for scientific purposes was permitted by local legislation. Professor Sławomir Podlaski, plant physiology major of Warsaw University of Life Sciences participated in the identification of specimens. The voucher specimen has not been deposited in any publicly available herbarium.

### Material

The research was carried out in 2013–2015. The study subjects were sugar beet fruits from 2 lots of the Polish commercial variety *Janosik* that differ in the level of vigour. *Janosik* variety was registered in year 2010, it is a diploid variety in normal type. It is resistant to rhizomania, has no tendency to bolting, develop high root and technological sugar yield, has low content of molasses and good processing properties. The average germination rate of seeds at 10 °C and 15 °C was assumed as a criterion of vigour in an optimum filter paper humidity of 60% full water capacity (FWC). Both fruit lots were characterized by a germination ability of 93 to 99% after 14 days (Table 1). Seeds were grown and produced in Po Valley (Italy). Harvested and dried seeds were subjected to pre-cleaning. Then seeds were transported to Poland and subjected to further stages of seed treatments. Each year the seeds were stored in cotton bags at room temperature and humidity. Fruit humidity varied in range of 8–9%. The characteristics of control fruits polished and rinsed in water collected in the years 2013–15 are presented in Table 1.

**Table 1:** The characteristics of control fruits

Fruits properties	Years of seed reproduction					
	2013		2014		2015	
	H	L	H	L	H	L
GA 4th day [%] 10 °C	18.2 ± 1.45	0	2.2 ± 0.18	1.8 ± 0.11	2.5 ± 0.19	0.5 ± 0.02
GA 14 th day 10 °C [%]	99.0 ± 3.60	96.2 ± 4.2	96.1 ± 2.10	93.0 ± 3.81	98.0 ± 2.1	92.8 ± 3.5
MTG 14th day [days]	5.6 ± 0.23	6.9 ± 0.35	6.2 ± 0.41	7.1 ± 0.34	6.2 ± 0.45	7.4 ± 0.61
Electric conductivity of fruits water extracts [mS cm^− 2^]	1.65 ± 0.25	2.44 ± 0.35	1.8 ± 0.12	3.0 ± 0.45	1.89 ± 0.13	2.9 ± 0.15
Total porosity [%]	48.1 ± 3.25	43.9 ± 2.56	47.0 ± 2.89	44.7 ± 0.19	46.2 ± 3.14	44.9 ± 2.45
Mesospore diameter [nm]	5.0 ± 0.32	1.0 ± 0.14	2.0 ± 0.14	1.0 ± 0.09	2.0 ± 0.08	1.0 ± 0.07

H, L – high and low level of seed vigour respectively

The best seeds with high vigour were obtained in 2013. They were characterized by high germination ability after 4 days, fast germination, lowest electric conductivity of fruit water extracts, relatively high total porosity and the largest mesopore diameter.

Before starting the tests, the fruits (including control combination) were polished and rinsed in water at 20 °C for 2 h, in accordance with the Kutnowska Hodowla Buraka Cukrowego Sp. z o. o. (KHBC) technology. KHBC is state-owned (polish) sugar beet breeding company.

Two seed priming technologies were used: Quick Beet (QB) (Podlaski S., Wzorek H., Chrobak Z., *Polish patent*, PL 207240, June 29th, 2005) and Quick Beet-1 (QB-1) (Podlaski S., Wzorek H., Chrobak Z., *Polish patent*, PL 216893, April 1st, 2011). Both methods used in this study are based on solid matrix priming (SMP) and use of zeolites as water carriers. The seeds are mixed with sodium zeolite NK 10 CECA in a specific ratio for a certain among of time, then separated on special sieves and allowed to dry.

### Physical properties of the pericarp

The physical properties of the pericarp were assessed after removing the operculum and seeds in the Department of Ceramics and Refractories at the AGH University of Science and Technology. The following physical parameters were determined: the helium density (helium pycnometer ACCuPyc 1330) which determines the density of the material with no pores determined on the basis of the material volume measurement; the apparent density, which is the ratio of the weight of the pericarp to its volume with pores; the total porosity, (GeoPyc 1360, density analyzer) which is the ratio of the volume of all pores to the total volume and the mezospore diameters (ASAP 2010 System) [23]. All instruments were obtained from Micrometrics Company, USA.

Helium density measurements were carried out using 10 helium flushes and 5 replicates of volume determination. The samples were dried at 105 °C before measurements and their mass was approximately 1.4 g. For apparent density, total porosity and total pore volume measurements the 0.36 g samples of pericarp were dried at 105 °C before measurements.

Before adsorptive measurement, the 1 g samples of pericarp were degassed at 105 °C Adsorption measurements were made at liquid nitrogen temperature using nitrogen as the adsorbate. Based on the measurements, specific surface area of materials (S_BET_) and mesopores volume were evaluated.

### Water potential and water content of the pericarp and seeds

The water potential of the seeds and pericarp was determined using a Dew Point Microvoltmeter HR-33 T and a C-52 measuring chamber (Wescor Inc., USA) after 2, 8, 24 and 48 h after placing the seeds on moist filter paper at a temperature of 15 °C and a filter paper humidity of 60% FWC. The tests were carried out in 5 replicates, and the incubation period of the seeds and pericarp in the measuring chamber was 15 min. The seeds were manually removed from the pericarp.

The water content of the seeds and pericarp was also determined after 2, 8, 24 and 48 h after placing the seeds on moist filter paper at a temperature of 15 °C and a filter paper humidity of 60% FWC. The material was dried at a temperature of 105 °C for 6 h, in 4 replicates. The results are expressed as the percentage of water in fresh or dry mass.

### Water diffusivity coefficient of the pericarp

By assuming that seeds have more or less a spherical shape, that water diffusion occurs only through the basal pore and that seeds do not change volume inside their cavity, the Crank diffusion equation can be used [9].

For estimation of water diffusion coefficient, calculations of the seed and fruit radius were necessary. It was assumed, that radius of each fruit to be half seed thickness, plus thickness of pericarp in place of basal pore (place of water entry). Half the length of the seed after extraction from seed cavity was taken as the seed radius. All measurement was carried out using a special micro meter made by the Agrophysics Institute in Lublin.

To determine the water diffusivity coefficient, the seeds were germinated on Petri dishes oat a temperature of 15 °C and a filter paper humidity of 60% FWC. After 6, 8, 24 and 48 h of germination, the water content in the pericarp and seeds was determined. The water diffusivity coefficient determines the amount of diffused water per unit area per unit of time. The greater the difference in the water content between the pericarp and the seed at a given time is, the lower the water diffusivity coefficient. The tests were carried out in three replications.

### Electrical conductivity of the water extracts

Three grams of fruit were mixed with deionized water equal to 10 times the volume of the fruit. After 2 h of stirring, the electric conductivity of the solution was measured using a Conductivity Meter CPC-505 (Elmetron) with EC − 60 electrode (Elmetron). The tests were carried out in three replications.

### Assessment of seed germination ability (GA) and germination speed (GS)

The seeds were germinated in accordance with the general standard of ISTA for sugar beets PN-79/R-65950 in 3 replications of 100 seeds each. Germination was conducted in plastic (ABS) boxes dimensions 220x130x40 mm under optimum moisture conditions on a filter paper, double pleated strips (2 seeds in a row), grade 3014, nominal thickness 0.22 μm, nominal weight 113 g m^− 2^ (Whatman) and humidity of 60% FWC (24 ml of water) at temperatures of 10 °C and 15 °C. Seeds orientation was random. Germination took place in complete darkness in Versatile Environmental Test Chamber MLR-350 (Sanyo).

Germination speed (GS) was estimated in the form of mean time to germinate (MTG) according to the formula MTG = Σ(n_i_ x d_i_)/N, where n_i_ is the number of germinated seeds at day i, d_i_ is the incubation period in days and N is the total number of germinated seeds.

### Statistical analysis

The obtained data were examined by Standard Error (SE), analysis of variance (*ANOVA*), and *Tukey*’s test (*p* < 0.05) (STAGRAPHICS Centurion version 18.1.06, 64-bit). Analysis of correlation and regression [35] were used to evaluate the relationship between the pairs of traits. In the tables presented hereafter, different *capital* and *small letters* denote the difference at *p* < 0.05 in the rows and columns, respectively.

## Results

The assessment of the physical properties of the pericarp is presented in Table 2. The obtained results were characterized by high variability caused by the natural variability of fruit characteristics and difficulties in extracting seeds from fruits.

**Table 2:** Physical properties of the pericarp, mean values from 3 years

Pericarp properies	Level of vigour	Seed treatment		
		Control	QB	QB − 1
Helium density [g cm^−3^]	H	1.16Aa	1.16Aa	1.15Aa
	L	1.31Ab	1.30Ab	1.28Ab
Apparent density [g cm^**−3**^]	H	0.62Aa	0.60Aa	0.60Aa
	L	0.70Ab	0.70Ab	0.70Ab
Total pore volume [cm^**3**^ g^−1^]	H	0.77Ab	0.79Ab	0.80Ab
	L	0.65Aa	0.63Aa	0.64Aa
Total porosity [%]	H	47.1Ab	47.8Ab	48.3Ab
	L	44.5Aa	45.0Aa	45.3Aa
Mesopore diameter [nm]	H	3Aa	5Bb	20Cd
	L	1Aa	3Ba	12Cc

H, L - high, low vigour respectively

Seed priming within each level of vigour did not significantly affect helium and apparent density, total pore volume and total porosity. Although no significant differences were found in total porosity depending on seed priming, tendency to increase porosity under the influence of priming could be observed. This is probably why seed priming significantly increased mesopore diameter in both high and low vigour seeds. In porous material such as pericarp, seeds are easiest to absorb water from mesopores, especially when they have a high water potential at the end of germination.

Low seed vigor was significantly associated with an increase in helium and aparent density and a decrease in total pore volume and mezospore diameter. It can be assumed that this could negatively affect the flow of water through the pericarp and possible wasching out inorganic germination inhibitors.

Effect of different seed treatment methods after 2, 8, 24 and 48 h since germination test start on water content and water potential of pericarp and seeds are presented in Tables 3 and 4 respectively.

**Table 3:** Changes in the water content [%] of the pericarp and seeds after 2, 8, 24 and 48 h since germination test start at 15 °C at 60% FWC. Average results from 3 years of experiments and 2 levels of seed vigour

Material	Seed treatment	Water content [%]			
		Time since germination test start [h]			
		2	8	24	48
Pericarp	Control	23.4 ± 1.0	25.4 ± 0.51	36.5 ± 0.90	43.7 ± 1.25
	QB	20.0 ± 1.23	30.9 ± 0.45	38.9 ± 1.12	48.9 ± 1.10
	QB-1	22.6 ± 1.26	29.8 ± 0.56	38.8 ± 1.06	44.8 ± 1.07
Seeds	Control	8.2 ± 0.26	10.8 ± 0.38	21.9 ± 1.09	31.6 ± 0.90
	QB	13.2 ± 0.50	13.8 ± 0.41	26.2 ± 0.57	35.8 ± 0.86
	QB-1	13.0 ± 0.41	13.6 ± 0.36	28.0 ± 0.93	37.0 ± 1.13

**Table 4:** Changes in the water potential [MPa] of the pericarp and seeds after 2. 8. 24 and 48 h since germination test start at 15 °C at 60% FWC. Average results from 3 years of experiments and 2 levels of seed vigour

Material	Seed treatment	Water potential [MPa]			
		Time since germination test start [h]			
		2	8	24	48
Pericarp	Control	−7.06 ± 0.51	−3.77 ± 0.31	−1.65 ± 0.12	−1.07 ± 0.21
	QB	−6.06 ± 0.37	−1.94 ± 0.25	−1.16 ± 0.22	−1.13 ± 0.21
	QB-1	−6.59 ± 0.41	−1.74 ± 0.28	−1.15 ± 0.18	−0.93 ± 0.18
Seeds	Control	−10.51 ± 0.40	−8.69 ± 0.35	−3.19 ± 0.22	−2.39 ± 0.35
	QB	−9.79 ± 0.22	−7.34 ± 0.20	−2.83 ± 0.15	−2.35 ± 0.23
	QB-1	−10.45 ± 0.42	−3.79 ± 0.31	−2.69 ± 0.23	−2.14 ± 0.25

As the germination proces advanced in subsequent time intervals, the pericarp and seeds water content increased gradually (Table 3). For the period from 8 to 48 h significant differences were found in water content of the pericarp control and primed seeds.

After 8, 24 and 48 h the pericarp from the primed seeds was always characterized by a higher water content (1.1 to 5.5%) than that of the control seeds.

Similarly, as in the case of the pericarp, the water content of the seeds increased as the germination process progressed. Primed seeds in each time interval were characterized by a higher water content ranging from 2.8 to 6.1%. A particularly significant difference in seed water content of 6.1 and 5.4% occurred between the fruits primed by the QB-1 method and the control fruits after 24 and 48 h since germination test start.

The water potential of seeds was lower than that in the pericarp in the same time interval (Table 4). After 8 and 24 h since germination test start, the water potential of the pericarp of primed fruits was always higher than in control fruits.

In the period of 24 to 48 h of germination, the increase in the water potential was slower than before and amounted to − 0.58 MPa for the pericarp and − 0.80 MPa for the control seeds.

During the germination period from 2 to 48 h, seed priming, especially with the QB-1 method, significantly increased the water potential of the seeds and pericarp. A particularly significant increase in the water potential of the pericarp and seeds occurred between 2 and 8 h of germination. During this period, the water potential of the control seeds increased by 1.82 MPa, while the water potential of the seeds primed by the QB-1 method increased by 4.85 MPa. Similarly, in the case of the control seed pericarp, the increase in the water potential was 2.29 MPa and that in the QB-1 priming method pericarp was 6.66 MPa.

After 24 h since germination test start the water content of the pericarp from high vigour control fruits was significantly lower (8.4%) than that of the pericarp from low vigour seeds (Table 5). Reverse relations occurred in the case of seeds. The water content of seeds from high vigour control fruits was significantly higher (5.4%) than that of the low vigour seeds.

**Table 5:** Comparison of the water content and water potential of the pericarp and seeds depending on the vigour level after 24 h of germination. Average results from 3 years of experiments

Material	Fruit treatment	Water content [%]		Water potential [MPa]	
		High vigour	Low vigour	High vigour	Low vigour
Pericarp	Control	32.3 ± 0.52	40.7 ± 0.53	−1.41 ± 0.15	−1.89 ± 0.10
	QB	37.9 ± 0.91	39.9 ± 0.85	−1.09 ± 0.12	−1.23 ± 0.09
	QB-1	38.1 ± 0.41	39.5 ± 0.52	−1.05 ± 0.11	−1.25 ± 0.08
Seeds	Control	24.6 ± 0.61	19.2 ± 0.32	−2.83 ± 0.15A	−3.55 ± 0.14
	QB	30.1 ± 0.47	22.3 ± 0.55	−2.50 ± 0.12A	−3.09 ± 0.15
	QB-1	32.4 ± 0.39	23.6 ± 0.57	−2.31 ± 0.13A	−3.07 ± 0.11

Differences in the water content of the pericarp were not apparent in the water potential value. The pericarp from low vigour fruits was characterized by a lower water potential compared to the pericarp from seeds with a higher water potential. A lower water content (5.4–8.8%) of low vigour seeds was also associated with a lower (− 0.59 and − 0.76 MPa) water potential. Considering values for the two levels of seed vigour, priming significantly increased the water content and water potential of the seeds compared to the control.

Moreover, seed priming resulted in a reduction in the differences between the water content of the pericarp with low and high vigour in relation to the control. The differences in the water content of the pericarp from the control fruits were 8.4% and those in the primed pericarp were 2.0 and 1.4%. Along with the decreases in the differences in water content of the pericarp from the seeds with two levels of vigour, the differences in the seed water content increased. The water content of the control seeds with a higher vigour was 5.4% higher than the water content of the seeds with low vigour. The seeds with a higher vigour primed by the QB and QB-1 methods were 6.8 and 8.8% higher than the water content of seeds with low vigour, respectively.

**Fig. 1:**
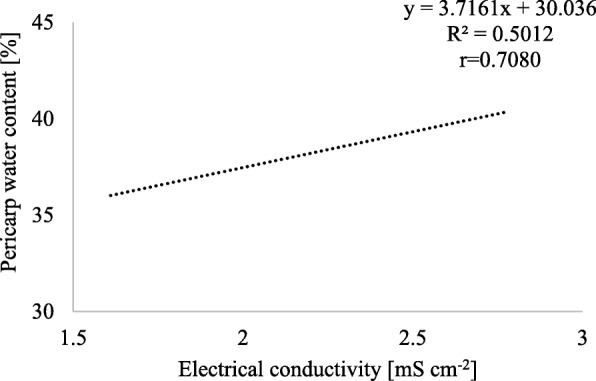
Relationship between the water content of the pericarp after 24 h since germination test start and the average electrical conductivity of the water extracts from control and primed fruits of high and low vigour seeds

**Fig. 2:**
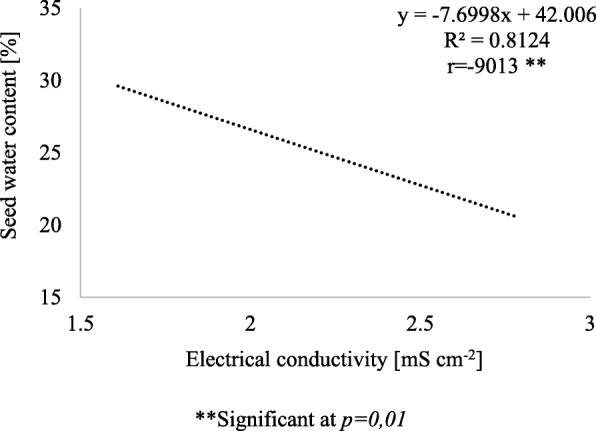
Relationship between average water content of the seed after 24 h since germination test start and the electrical conductivity of the water extracts from control and primed fruits of high and low vigour seeds

The level of seed vigour had a significant effect on the electrical conductivity of fruit water extracts (Table 6). Seeds with low vigour were characterized by 58% higher electrical conductivity than seeds with high vigour. For 2 levels of vigour, seed priming caused a significant reduction in the electrical conductivity of aqueous extracts. Therefore, seed priming may directly affect the water content of the seeds and pericarp (Figs. 1 and 2) and the water diffusivity coefficient of the pericarp (Table 7).

**Table 6:** Effect of two levels of seed vigour on the electric conductivity of fruit extracts [mS cm^− 2^]. Average results from 3 years of experiments. Fruits were polished and rinsed in water at 20^0^ C for 2 h

Fruit treatment	Level of seeds vigour	
	High	Low
Control	1.78 ± 0.05	2.78 ± 0.08
QB	1.64 ± 0.04	2.63 ± 0.08
QB-1	1.61 ± 0.05	2.52 ± 0.07

**Table 7:** Water diffusivity coefficient of the pericarp during seed germination [cm^2^ d^− 1^] at 15 °C and 60% FWC

Fruit treatment	High vigour seeds			Low vigour seeds		
	Time since germination test start [h]					
	6	24	48	6	24	48
Control	0.14 ± 0.01	0.14 ± 0.01	0.14 ± 0.008	0.08 ± 0.007	0.09 ± 0.01	0.10 ± 0.009
QB	0.15 ± 0.01	0.15 ± 0.01	0.16 ± 0.01	0.10 ± 0.008	0.11 ± 0.008	0.12 ± 0.008
QB-1	0.16 ± 0.009	0.17 ± 0.01	0.17 ± 0.01	0.11b ± 0.008	0.12 ± 0.01	0.12 ± 0.01

Figures 1 and 2 show that as the electric conductivity increases by 1 mS cm^− 2^, the pericarp water content increases by 3.7% and the seed water content decreases by 7.6%.

The water diffusivity coefficient determines the amount of diffused water per unit area per unit of time. Values of water diffusivity of pericarp for different seed treatments and 2 levels of vigour are shown in Table 7.

There was a significant difference in the value of the pericarp water diffusivity coefficient depending on the level of seed vigour. The pericarp water diffusivity coefficient of the low vigour seeds, at the same time intervals (6, 24 and 48 h) and the same fruit treatment was always lower than high vigour seeds.

The value of the pericarp water diffusivity coefficient was relatively constant in the period from 6 to 48 h of germination. Seed priming caused a significant increase in the average value of the pericarp water diffusivity coefficient in relation to control seeds by 0.01 and 0.03 cm^2^ d^− 1^.

Seeds belonging to both groups of vigour had a very similar germination ability of approximately 94–98%, after 14 days. In contrast, seeds with higher vigour were characterized by faster germination (0.39–0.99 d) than the seeds with low vigour (Table 8).

**Table 8:** Mean time to germinate [d] of seeds after 14 days, average results from 3 years of experiments at temperatures 10 and 15 °C

Fruit treatment	Level of seed vigour	
	High	Low
Control	4.78 ± 0.31	5.65 ± 0.38
QB	3.49 ± 0.28	4.48 ± 0.28
QB-1	3.35 ± 0.15	3.74 ± 0.20

On average, for 2 levels of seed vigour, both priming methods significantly accelerated seed germination, the QB method decreased the germination time by 1.29 (high vigour) and 1.17 days (low vigour) and the QB-1 method decreased the germination time by 1.43 (high vigour) and 1.91 days (low vigour).

The QB-1 priming method accelerated the germination of high vigour seeds by only 0.14 days, whereas priming method accelerated the germination of low vigour seeds by as much as 0.74 days in comparison to low vigour seeds.

**Fig. 3:**
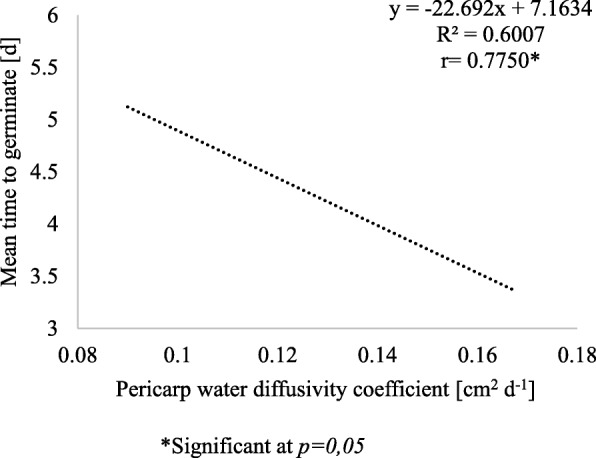
Relationship between the mean germination time at 10 and 15 °C after 14 d and the water diffusivity coefficient of the pericarp after 4, 24 and 48 h of germination. Average results from 3 years of experiments and 2 levels of vigour

Relationship between mean germination time (for 2 level of seed vigour and 3 ways of fruits treatment) and pericarp water diffusivity coefficient is presented in Fig. 3.

It is difficult to say to what extent the acceleration of germination was caused by the changes in the biological properties of the seeds or the physicochemical properties of the pericarp. A partial answer to this problem is shown in Fig. 3. The analysis of correlation and regression showed that the average value of the water diffusivity coefficient of the pericarp affects the average MTG value determined at temperatures of 10 and 15 °C. Increasing the average value of the water diffusivity coefficient of the pericarp by 0.01 cm^2^ d^− 1^ reduces the mean germination time by 0.2 days.

## Discussion

The data presented in this study indicate that sugar beet seed priming causes not only a relatively well known change in the physiological and biochemical properties of seeds ([5, 21, 36], Herman et al. 2007, [1, 11]) but also a modification in the properties of pericarp, facilitating the flow of water through the pericarp. The obtained results are arranged in a logical sequence of events showing the relationship between the water diffusivity of the pericarp and the germination rate of seeds.

Studies have not shown that fruit treatment significantly affected helium and apparent density, total pore volume and total porosity. However, a clear tendency to increase total porosity was observed under the influence of seed priming. This fact probably caused seed priming to increase the diameter of pericarp mesopores from 3 to 20 and from 1 to 2 nm for high and low vigor seeds respectively.

As a consequence, in the pericarp, the water potential increased particularly strongly after 8 h of germination. A similar phenomenon occurred in the case of the seeds. After 8 h of germination, the water potential of the seeds primed by the QB-1 method was 4.9 MPa higher than that in the control seeds. Subsequently, the difference was reduced, but until the germination process was completed, the primed seeds were characterized by a higher water potential than the control seeds. As a result, the seed water potential level of approximately − 1.0 MPa was reached quicker. According to Bradford [4], the radicle (root tip) protruding through and beyond the operculum appears at this level.

A lower level of seed vigour had a higher impact on the water content and water potential of the seeds and pericarp than higher vigour. At the same interval time, the low level of vigour increased the pericarp water content and decreased the water content in the seeds, whereas in the case of the water potential, the low vigour of the seeds was associated with the lower water potential value of the pericarp and seeds. Relationship between fruits water absorption and fruit maturity was also shown by Snyder [34] and Salini and Boelt [32] who observed a higher water content in immature fruits. The water content and water potential of the pericarp and seeds were correlated by the electrical conductivity of the aqueous extracts. This value could be a measured by the content of germination inhibitors in forms of inorganic salts. The content of these inhibitors depends mainly on the environmental conditions during seed maturation and maturity level of seed [26].

Electric conductivity of the seeds leachate can be used as vigour test for seeds of *Glycine max, Phaseolus vulgaris, Pisum sativum and Cicer arietinum* seeds [17].

Increased water content in the pericarp combined with a reduction in water content in the seeds is evidence of problems with the water flow through the pericarp, which is evidenced by a reduction in the value of the water diffusivity coefficient of the pericarp.

A comparison of average pericarp water diffusivity of the raw fruit (0.00134 cm^2^ d^− 1^) from 48 Polish sugar beet lines stock [27] and the diffusivity of pericarp commercial variety *Janosik* (0.10–0.15 cm^2^ d^− 1^) clearly indicates the importance of reproduction seeds in Po Valley (Italy) and proper seed treatment in form of polishing, washing, grading and priming. Similarly, Ignatz et al. [14] confirm that the combined polishing and washing treatment resulting in significant improvements of the germinations performance due to a combined removal of mechanical and inhibitor constraints.

The increase in the water content of the pericarp in fruits with low vigour was accompanied by a decrease in the water potential value in the pericarp. Most likely, the low water potential was caused by the formation of an osmotic solution coming from the dissolving crystals of chemical compounds. Studies by Podlaski [27] have shown that low vigour is usually accompanied by a higher content of chemical compounds in the pericarp, which gradually dissolve, creating an osmotic solution that inhibits water uptake.

In this study, correlations were found between the seed germination rate and the pericarp water diffusivity coefficient. From this statement follows the practical conclusion that the pericarp should be as thin as possible, should not contain crystals of inorganic chemical compounds and organic inhibitors and priming technology should stimulate the water flow through it.

## Conclusions

1. Priming sugar beet seeds by the SMP method significantly increased the mesopore diameter in pericarp.
2. The increase in mesopore diameter and total porosity increases the water content in the pericarp and the water potential value of the seeds.
3. The low vigour of seeds is connected with the higher content of inorganic chemicals in the pericarp, which corresponds to the higher electrical conductivity of water extracts.
4. Seed priming and a higher level of vigour are associated with a higher value of the water diffusivity coefficient of the pericarp.
5. A higher value of the water diffusivity coefficient of the pericarp accelerates seed germination.

## Data Availability

The datasets used and/or analysed during the current study available from the corresponding author on reasonable request.

## Abbreviations

ABA: Abscisic acid
ABS: Acrylonitrile butadiene styrene
AGH: University of science and technology
FWC: Full water capacity
GA: Germination ability
GS: Germination speed
ISTA: International seed testing association
KHBC: Kutnowska sugar beet breeding company
MTG: Mean time to germinate
QB: Quick Beet
QB-1: Quick Beet-1
SE: Standard error
SEM: Scanning electron microscopy
SMP: Solid matrix priming
